# Supplementary figures and images for: MPL36, a major plasminogen (PLG) receptor in pathogenic Leptospira, has an essential role during infection
Source: PLoS Pathog. 2023 Jul 24;19(7):e1011313. doi: 10.1371/journal.ppat.1011313 (PMC10399853; doi:10.1371/journal.ppat.1011313)

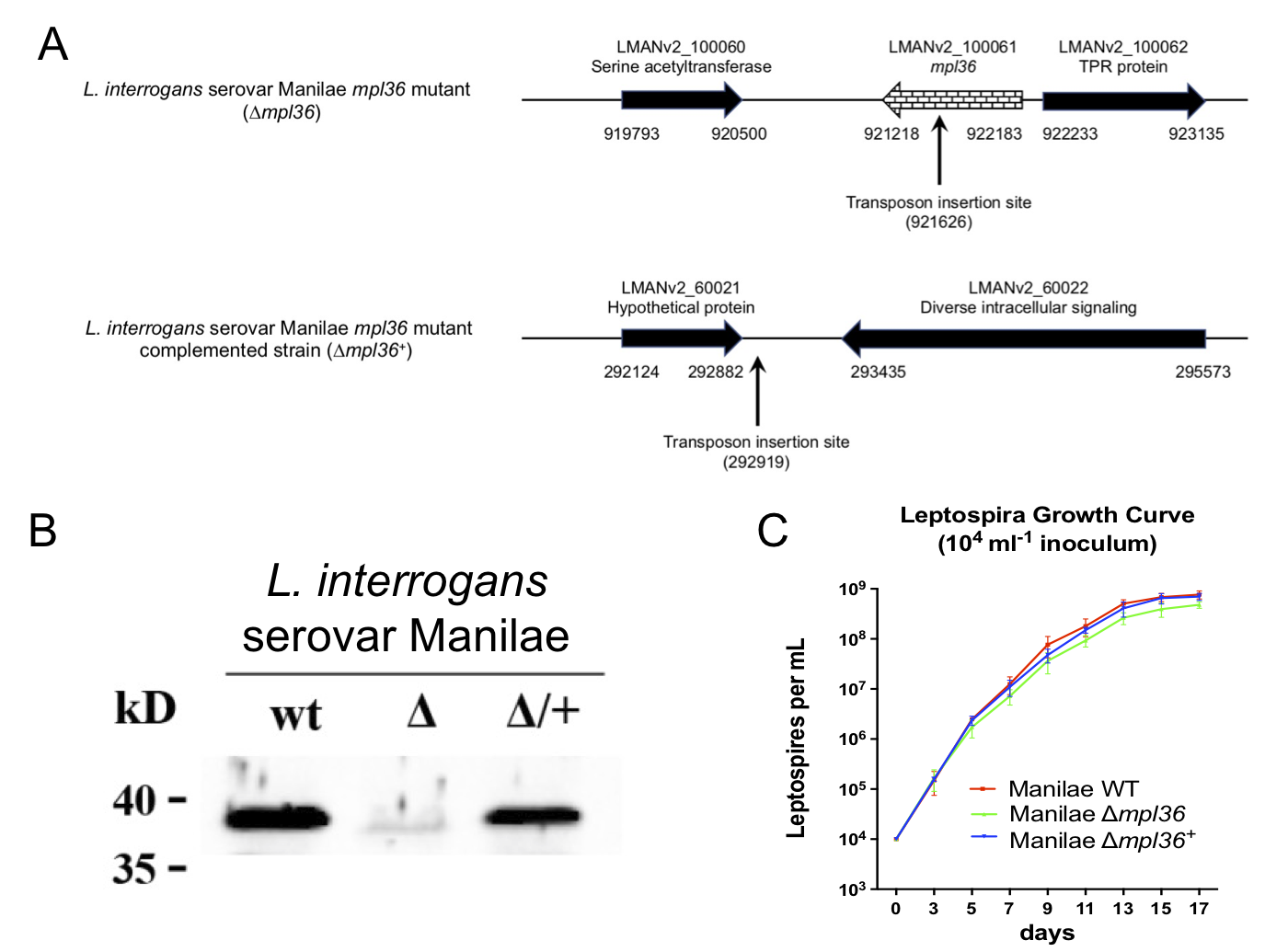

Supplement: S1 Fig — (A) Schematic representation of Himar1 transposon insertion positions in L. interrogans Manilae L495. The insertion sites of the transposon in the chromosome of the mpl36 gene in strain Manilae WT, and the insertion site of the transposon containing the spectinomycin resistance cassette and mpl36 gene for complementation are indicated. (B) Immunoblot analysis of Manilae WT, mutant Δmpl36 and complemented strain Δmpl36+ using a rabbit polyclonal antibody against rMPL36. The visible band has a molecular weight of ~40 kDa, which is in accordance with the predicted molecular weight of MPL36. The mutant strain lacks the expression of the protein. Molecular mass markers are shown on the left. (C) Motility assay results for Manilae WT, Δmpl36, and Δmpl36+ strains at 30°C. Bacteria (105 cells) were inoculated on 0.5% agarose EMJH plates (each square, 1 cm2) for 10 days. (D) Growth curve analysis of Manilae WT, Δmpl36, and Δmpl36+ strains at 30°C. Bacteria were grown in EMJH medium without agitation, and the counting was performed using dark field microscopy. Results represent the average ± the standard deviation of three independent experiments. (TIFF) [file ppat.1011313.s001.tiff]

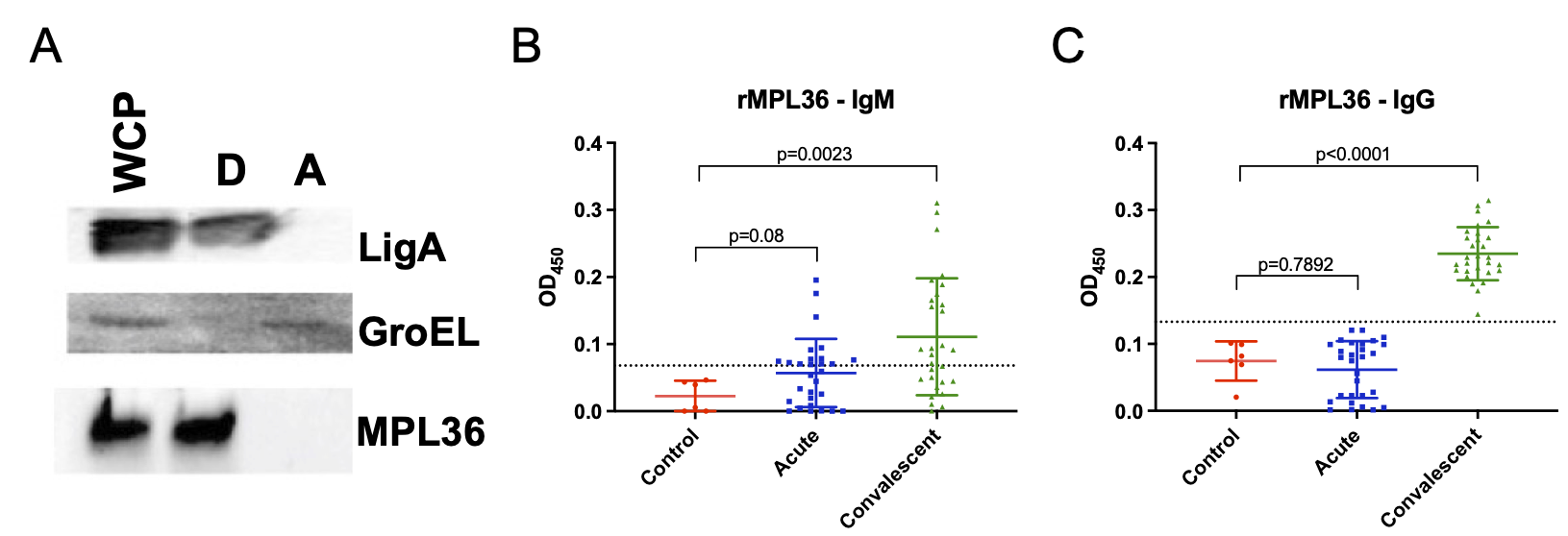

Supplement: S2 Fig — (A) Whole intact Manilae WT (WCP) was treated with Triton X-114 for phase partitioning of Leptospira membrane proteins. Immuno-blot analysis was conducted with detergent (D) and aqueous (A) phase using polyclonal rabbit antisera against LigA (outer membrane), GroEL (cytoplasmic), and MPL36 proteins. Antibodies to rMPL36 in human sera from individuals with confirmed severe leptospirosis were measured by an ELISA assay. Reactivity of the rMPL36 with acute and convalescent serum samples was tested for IgM (B) and IgG (C) levels separately. The dashed line represents the threshold calculated based on 2.5 SD of the average OD signal of sera from healthy US individuals used as control. Data show the mean absorbance value at 450 nm ± the standard deviation of all individuals tested. (TIFF) [file ppat.1011313.s002.tiff]

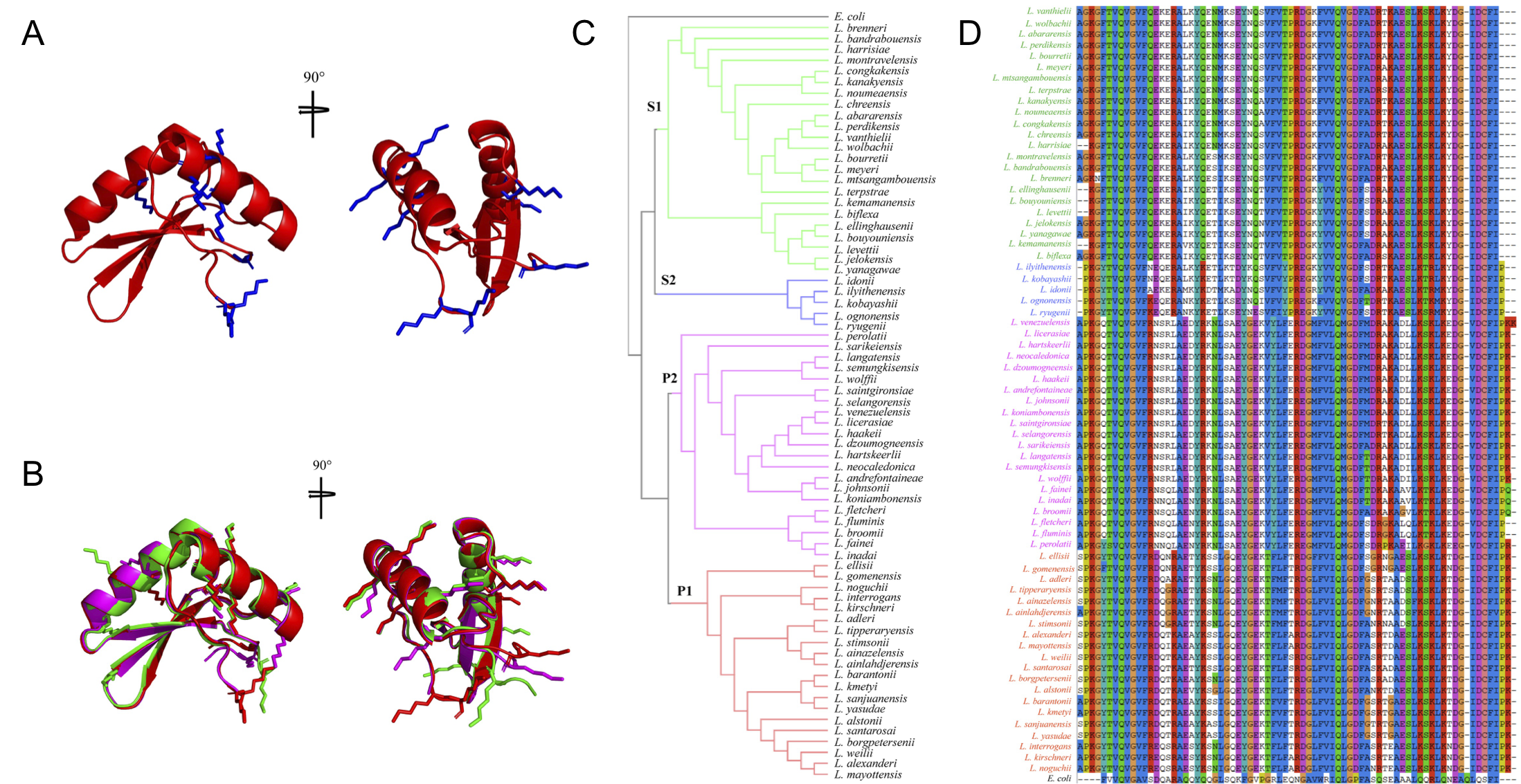

Supplement: S3 Fig — (A) Tertiary predicted model of the SPOR domain of MPL36 in L. interrogans, visualized by PyMOL, showing the exposed lysine residues in blue. (B) Alignment of tertiary predicted structures of SPOR domain from L. interrogans (P1-red), L. fainei (P2-pink), and L. biflexa (S1-green), visualized by PyMOL, showing the exposed lysine residues. (C) Dendrogram resulting from multiple alignments performed by ClustalW of SPOR domain of MPL36 with other similar sequences of all 69 Leptospira species separated by groups: P1 (red), P2 (pink), S1 (green), and S2 (blue), identified by BLASTp. (D) Alignment of the SPOR domain of MPL36 with other similar sequences of all 69 Leptospira species: P1 (red), P2 (pink), S1 (green), and S2 (blue). Alignment was done using the ClustalX software and similar amino acids have the same colors. (TIFF) [file ppat.1011313.s003.tiff]

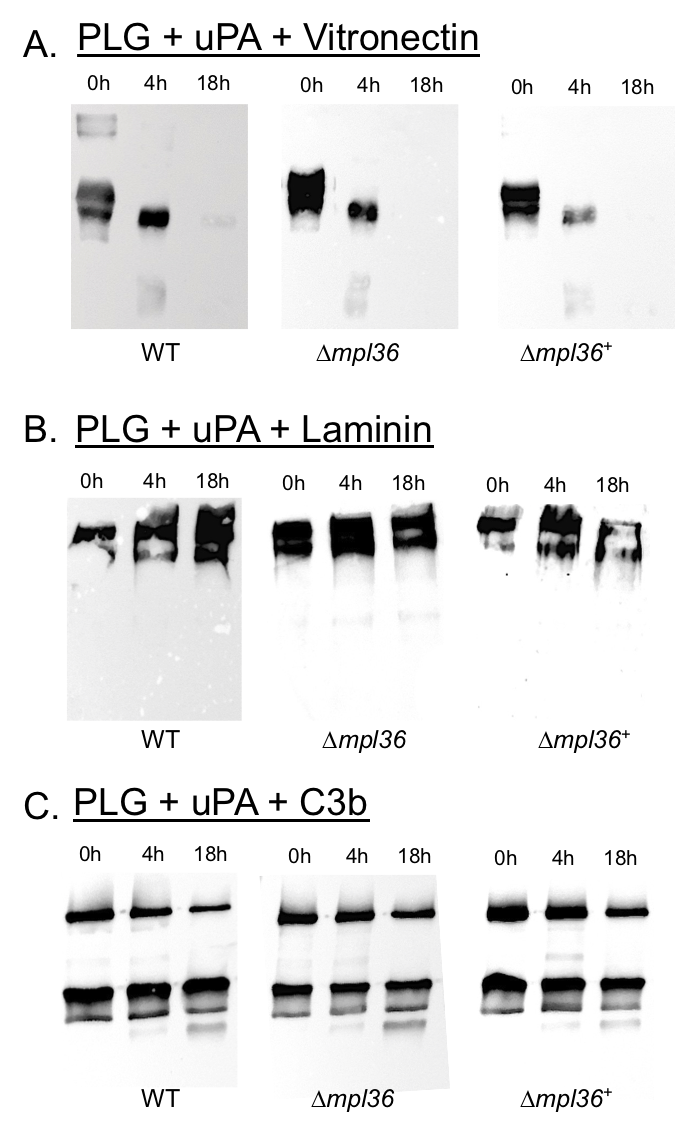

Supplement: S4 Fig — Strains Manilae WT, Δmpl36, Δmpl36+ (108 cells) were incubated with purified human PLG (10 μg). After washing, laminin (5 μg), vitronectin (2.5 μg) or C3b (1.5 μg) plus uPA (3 U) were added and incubated for up to 18 h. Leptospiral supernatants were collected and analyzed by western blot using anti-human vitronectin, laminin, or C3b (1:5,000) followed by peroxidase-conjugated secondary antibodies (1: 10,000). (TIFF) [file ppat.1011313.s004.tiff]

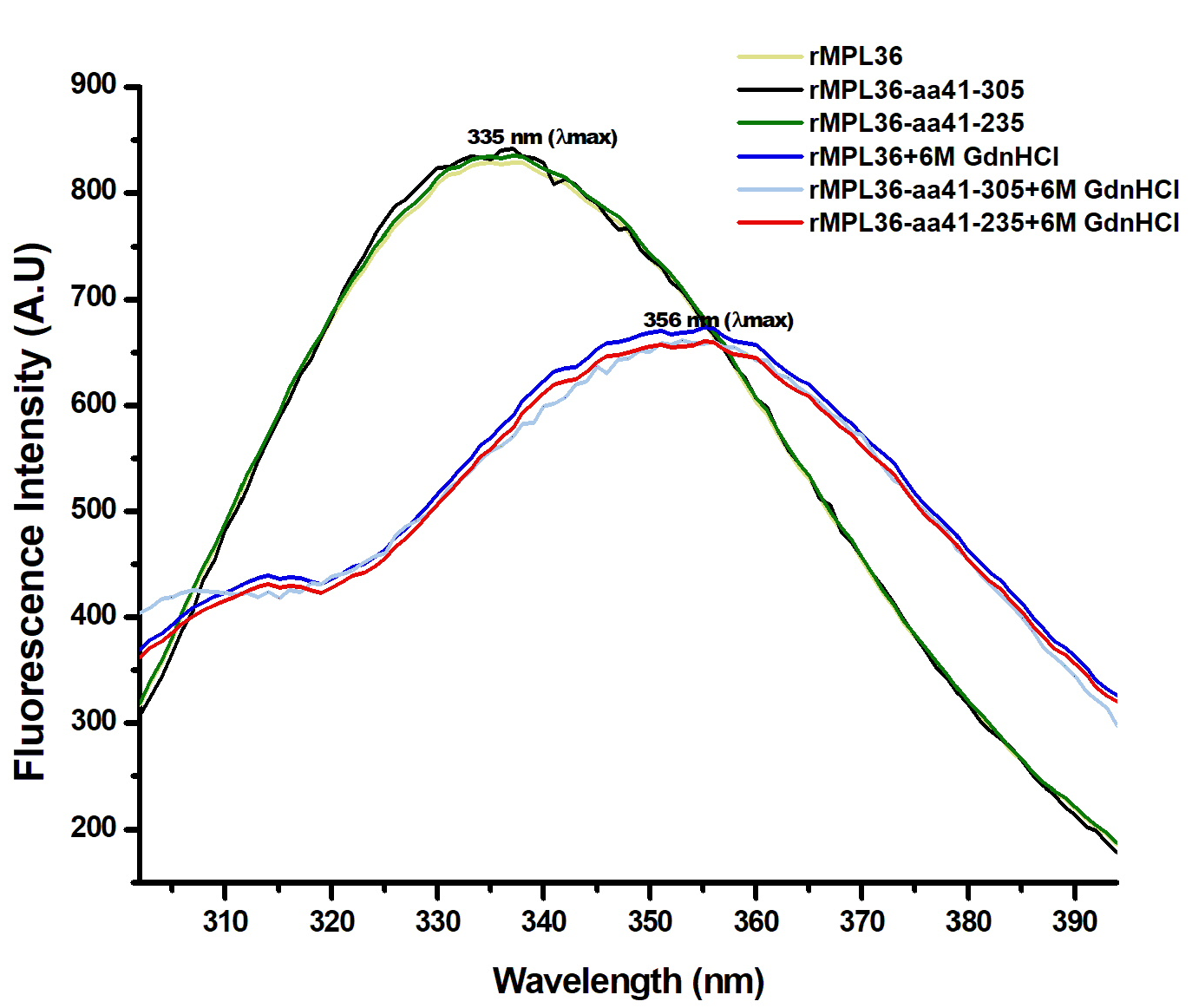

Supplement: S5 Fig — Tryptophan fluorescence emission spectra of rMPL36, rMPL36 aa41-305, rMPL36 aa41-235, and the corresponding unfolded proteins. Fluorescence measurements were carried out on a fluorescence spectrophotometer in a 1 cm path length rectangular quartz cuvette. The intrinsic fluorescence emission of native and unfolded proteins was measured in 10 mM PBS, with excitation at 280 nm and emission recorded in the range of 300–400 nm. (TIFF) [file ppat.1011313.s005.tiff]
